# Hospital Acquired Sepsis, Disease Prevalence, and Recent Advances in Sepsis Mitigation

**DOI:** 10.3390/pathogens13060461

**Published:** 2024-05-30

**Authors:** Mary Garvey

**Affiliations:** 1 Department of Life Science, Atlantic Technological University, F91 YW50 Sligo, Ireland; mary.garvey@atu.ie; Tel.: +353-0719-305-529; 2 Centre for Precision Engineering, Materials and Manufacturing Research (PEM), Atlantic Technological University, F91 YW50 Sligo, Ireland

**Keywords:** sepsis, immune dysregulation, mortality, neonates, resistance, biomarker

## Abstract

Sepsis is a life-threatening organ dysfunction caused by a dysregulated host response to infection, commonly associated with nosocomial transmission. Gram-negative bacterial species are particularly problematic due to the release of the lipopolysaccharide toxins upon cell death. The lipopolysaccharide toxin of *E. coli* has a greater immunogenic potential than that of other Gram-negative bacteria. The resultant dysregulation of the immune system is associated with organ failure and mortality, with pregnant women, ICU patients, and neonates being particularly vulnerable. Additionally, sepsis recovery patients have an increased risk of re-hospitalisation, chronic illness, co-morbidities, organ damage/failure, and a reduced life expectancy. The emergence and increasing prevalence of antimicrobial resistance in bacterial and fungal species has impacted the treatment of sepsis patients, leading to increasing mortality rates. Multidrug resistant pathogens including vancomycin-resistant Enterococcus, beta lactam-resistant *Klebsiella*, and carbapenem-resistant *Acinetobacter* species are associated with an increased risk of mortality. To improve the prognosis of sepsis patients, predominantly high-risk neonates, advances must be made in the early diagnosis, triage, and control of sepsis. The identification of suitable biomarkers and biomarker combinations, coupled with machine learning and artificial intelligence, show promise in early detection protocols. Rapid diagnosis of sepsis in patients is essential to inform on clinical treatment, especially with resistant infectious agents. This timely review aims to discuss sepsis prevalence, aetiology, and recent advances towards disease mitigation and control.

## 1. Introduction

Sepsis is resultant from an excessive response of a person’s immune system following contact with an infectious agent or their toxins or endogenous agents including cytokines, i.e., interleukins (IL). By definition, sepsis is a life-threatening organ dysfunction caused by a dysregulated host response to infection [1]. The identification and correct, timely management in the initial hours after development of sepsis improves outcomes [2]. Gram-negative bacteria (GNB), Gram-positive bacteria (GPB), and fungal pathogens are associated with nosocomial sepsis, while microbial species including *Staphylococcus aureus* (47.3%), *Enterococcus* species (10.8%), and *Candida* species (10.1%) have all been identified in sepsis cases [3]. Bacterial cell wall components including the lipopolysaccharide (LPS) of GNB, peptidoglycan (PGN), and lipoteichoic acid (LTA) of GPB act as pyrogenic material [3]. By the latest definition, sepsis is a “life-threatening organ dysfunction caused by a dysregulated host response to infection”, with the organ dysfunction being allocated a sequential organ failure assessment (SOFA) score [4]. The early definition of sepsis based on the initiation of a systemic inflammatory response syndrome (SIRS) lacks sensitivity and specificity, while organ dysfunction is not a specific indicator of infection or sepsis. The definition of sepsis therefore has changed three times since 1991 with no gold standard definition clinically in use. As per the third definition of sepsis, the SOFA was used to measure organ dysfunction due to its simplicity with the quick SOFA (qSOFA) tool, as an indicator of patients that were not in the ICU but were likely to develop sepsis as determined from the retrospectively derived databases [5]. The dysregulation of the immune system can result in organ failure and death, with pregnant women, patients in intensive care units (ICUs), and neonates being particularly vulnerable. The organs most affected by sepsis include the kidneys, liver, lungs, heart, central nervous system (CNS), and hematologic system [4]. Sepsis affects ca. 49 million people with 11 million deaths annually, worldwide, causing the WHO to list sepsis as a global health priority [6]. Studies show a sepsis mortality rate of 40% in ICUs and ca. 25% in hospitals (Table 1) [7]. Approximately one-third of neonatal deaths are due to sepsis, with GNB being predominately associated with 30% of these deaths that are thus directly related to the presence of antimicrobial resistant (AMR) species [8]. Studies show that sepsis associated with GNB is more severe than that of GPB, with increased levels of inflammatory components [3]. Furthermore, the LPS toxin of *E. coli* has a greater immunogenic potential than that which is excreted by other GNB [9]. Studies describe the LPS concentration released by ESBL *E. coli* following exposure to the bactericidal antibiotic, ceftazidime, in a dose-dependent manner [10]. Risk factors associated with sepsis mortality include age, co-morbidities, bloodstream infections (BSIs), immune suppression therapy, invasive surgeries, medical devices, and AMR pathogens [7]. Lactic acid levels in septic shock patients are a predictor of mortality with higher levels associated with increased rates of patient death [11]. A pre-existing immunocompromise status is a significant risk factor for sepsis, with neonates at high risk [12]. In critically ill patients, structural alterations and protein imbalances lead to systemic muscle wasting, mitochondrial dysfunction, loss of muscle membrane excitability and ion channel issues [13]. While mortality rates are decreasing from 50% prior to 2000 to ca 25% currently, the incidence of sepsis continues to increase with better diagnosis and treatment strategies in clinical settings [14]. Importantly, however, ca. 75% of patients who have recovered from sepsis have higher rates of long-term morbidities, including mental health issues, cognitive difficulties, physical issues, and mortalities post discharge, termed “post-sepsis syndrome” (PSS) [14,15]. Sepsis recovery patients are therefore at an increased risk of re-hospitalisation, chronic illness, co-morbidities, organ damage/failure (kidney, heart and respiratory) and have a reduced life expectancy [16]. PSS results from immune system malfunction, chronic inflammation, oxidative stress, and dysfunction of the mitochondria [17]. Indeed, studies show that mortality 6 months post disease is ca. 60% for septic shock and ca. 36% for severe sepsis [18]. Current treatment options for sepsis include antibiotic therapy, surgery of damaged tissue and providing organ support according to the 2021 Surviving Sepsis Campaign (SSC) guidelines [19] (Table 1). European Union (EU) funded research (ImmunoSep) aims to restore the immune function in sepsis patients using personalised immunotherapy and a next-generation theranostics platform [20]. With increasing prevalence of AMR infectious disease and difficult-to-treat pathogens, sepsis morbidity and mortality rates are also likely to increase. Furthermore, with the alarming rates of neonate fatalities, advances must be made in the mitigation and control of sepsis. This timely review aims to discuss sepsis prevalence, aetiology, in relation to the growing issue of antimicrobial resistance, and the recent advances towards disease mitigation and control.

## 2. Aetiology of Pathogenic Sepsis

Bacterial pathogens are the most frequent cause of infectious sepsis, with viral and fungal species also contributing to the incidence of disease, particularly in immunocompromised patients or patients with co-morbidities [12]. The immune response and sepsis characteristics are common regardless of the causative agent, i.e., bacterial, fungal, or viral. It is the immune status and inflammatory response of the patient which leads to morbidity and mortality. Host immunity is active in the development of sepsis and is activated following contact with a pathogen where specific receptors, termed pathogen recognitions receptors (PRRs), on the surface of immune cells are activated by contact with microbial pathogen-associated molecular patterns (PAMPs) on microbial cells [21]. PAMPs are present on all microbes with varying compositions, the LPS toxin and flagellin are associated with GNB, LTA with GPB, chitin, β-1,3-glucan, and β-1,6-glucan in fungal species and nucleic acids (RNA and DNA) with viral pathogens [22]. For example, the Gram-negative LPS binds with cell-mediated immune components, including cell-medicated CD 14, CD 16, CD 18, humoral-mediated antibodies and lactoferrin, and activates the Toll-like receptors (TLR) [23]. Sepsis resulting from the LPS toxin is associated with cardiovascular failure, organ damage and organ failure in patients [10]. Contact between immune cells and these PAMPs triggers pro- and anti-inflammatory processes, which result in the dysregulation of the innate and acquired immune systems and may lead to excessive inflammation, immune suppression, and a loss of immune homeostasis [9]. Damage-associated molecular patterns (DAMPs) include cellular proteins, nucleic acids, nuclear lipids, cytoplasm, mitochondria, and endogenous granules of the host that are also active in sepsis [24]. PRRs include TLRs, nucleotide-binding oligomerization-domain-like receptors, cytosolic RNA and DNA sensors, C-type lectin receptors, and Nod-like receptors (NLRs) amongst other types. These trigger a cascade activation of pro-inflammatory cytokines including interleukins (IL) IL1, IL 18, IL 6, chemokines, and growth factors involved in an excessive inflammatory response and immune dysregulation [21] or cytokine storm. Cytokines activate the macrophages and mononuclear cells (lymphocytes, monocytes, natural killer cells (NK cells), dendritic cells) to phagocytose invading pathogens, present antigens, and monitor infection [24]. Sepsis patients have a reduction in peripheral mononuclear cells, where mitochondrial injury and dysfunction also contributes to sepsis-induced organ damage. TLRs are particularly important mediators of sepsis, with TL4 being considered an essential molecule in innate immunity, mediating the inflammatory response to PAMPs and DAMPs generated during infection [25]. Circulating DAMPs, PAMPs, and pro-inflammatory cytokines activate the cardiac and endothelial cells, impacting cardiac function and disrupting pulmonary endothelial barriers, resulting in respiratory distress, disruption of the gastrointestinal (GIT) barrier and permeability, and may impact the blood–brain barrier’s permeability to toxins and cytokines thus leading to septic encephalopathy amongst other issues [26]. Septic encephalopathy is associated with higher ICU and hospital mortality rates, and long-term cognitive and functional issues in surviving patients [27]. The relationship between inflammation and coagulation is also a major factor in sepsis [28]. Inflammasomes, which are protein complexes produced following contact with PAMPs or DAMPs, regulate the secretion of pro-inflammatory interleukins and indue pyroptosis in conjunction with specific caspase and apoptosis associated protein complexes [29]. The studies of Deng et al. (2018) demonstrate the role of high mobility group box-1 protein excreted by the liver cells, which transports LPS toxins inside the cells via the receptor for advanced glycated end products (RAGE) pathway and triggers caspase-11 activation during bacterial sepsis [30]. The pro-inflammatory caspase 11 triggers inflammasome-mediated caspase-1 activation with pro-IL-1b cleavage and IL-1b release [30]. Importantly, caspase-1 has demonstrated a correlation with sepsis severity, where high caspase-1 activation in the first 24 h of sepsis is associated with increased mortality [31]. RAGE receptors and caspase-11 are present on endothelial cells and myeloid cells [32,33]. The inflammatory stage of sepsis may persist for days with mortality highest at ca. day 5, secondary infection and a reduced immune capacity contribute to deaths after 20 days [12,34,35]. Interestingly, the diversity of the resident GIT microbiota is disrupted in sepsis patients with a dysbiosis of key *Firmicute* and *Bacteroide* species, which have an impact on the immune response of the host, epithelial barrier function, and production of key regulatory molecules such as short chain fatty acids [9,36]. The relationship between the host GIT microbiota and numerous disease states has been well established [9,33].

**Table 1:** Clinical characteristics and mortality rates of each sepsis category.

Sepsis Category	Characteristics	Guidelines According to SSC 2021	Mortality
Sepsis	Life-threatening organ dysfunction caused by a dysregulated host response to infection. Organ dysfunction can be identified as an acute change in total SOFA score ≥ 2 points consequent to the infection [1,37]	30 mL/kg of IV crystalloid fluid within the 3 h of resuscitation, Lower serum lactate levels, monitor capillary filling to assess tissue perfusion, antibiotic therapy [19] Corticosteroids in patients in septic shock who require vasopressor therapy [19]	40% in ICUs and ca. 25% in hospitals
Septic shock	Subtype of sepsis, manifested by circulatory, cellular, and metabolic instability [1,19] vasopressor requirement to maintain a mean arterial pressure of 65 mm Hg or greater and serum lactate level greater than 2 mmol/L (>18 mg/dL) in the absence of hypovolemia as per sepsis 3 [5,19,34]		Average 30-day septic shock mortality of 34.7% and 90-day septic shock mortality of 38.5% [38] Ca. 45% [19]
Multi-organ dysfunction syndrome	MODS is the presence of altered organ function in an acutely ill patient such that homeostasis cannot be maintained without intervention	Sepsis-induced respiratory failure—high flow nasal oxygen (HFNO) over non-invasive ventilation.	With two organs affected the mortality is ca. 30%, with 3 or 4 organs affected the mortality will rise to 50–70% [39]

## 3. Hospital Acquired Sepsis

Pathogenic sepsis can be a hospital-acquired sepsis (HAS) resulting from hospital-acquired infections (HAIs) or community-acquired sepsis. Studies show that HAS results in longer ICU and hospital stays and increased mortality rates compared to community-acquired sepsis, with 30.7% versus 15.6%, respectively [40]. Approximately 24% of sepsis cases with organ dysfunction are acquired in the ICU, with ca. 49% being acquired in hospitals [41]. Studies have shown that 50% of sepsis-induced mortality is hospital-acquired sepsis (HAS), with the ICU admittance and AMR species contributing to disease severity and fatality [12]. Furthermore, the incidence of neonatal sepsis in higher in ICU-admitted infants [41]. HAIs are typically associated with medical devices, e.g., catheter-associated urinary tract infections, central line-associated bloodstream infections (BSIs), surgical site infections, ventilator-associated pneumonia (VAP) [9]. Research has shown that indwelling medical devices are associated with increased risk of sepsis and sepsis prognosis as well as longer hospital stays [42]. Hospital-acquired pneumonia and *Clostridium difficile* infections are also nosocomial in nature [43]. Disease transmission in hospital settings is associated with horizontal transmission, i.e., patient to patient or staff to patient, fomites in the hospital environment such as beds and medical devices, particularly the reusable ones. In terms of HAIs, the WHO bacterial and fungal priority pathogen lists are highly relevant and detail critically and highly important pathogens displaying high levels of AMR. The ESKAPE (*Enterococcus faecium*, *Staphylococcus aureus*, *Klebsiella pneumoniae*, *Acinetobacter baumannii*, *Pseudomonas aeruginosa*, and *Enterobacter* species) pathogens and fungal priority pathogens, e.g., *Candida*, *Aspergillus*, and *Cryptococcus* spp., have high mortality rates coupled with multidrug resistance (MDR) and extensive drug resistance (XDR) [44].

### 3.1. Bacterial Sepsis

The GPB methicillin-resistant *Staphylococcus aureus* (MRSA), and *P. aeruginosa* amongst the other GNB, are frequently causative of hospital-acquired pneumonia associated with intubation or VAPs in ICUs, having significant mortality rates [45,46]. Sepsis, however, is most associated with BSIs resultant from improperly sterilised or contaminated medical devices including catheters, intravenous lines, and mechanical ventilators and is a major complication in ICU patients [9]. Approximately 60% of primary BSIs are caused by Gram-positive ESKAPE pathogens, namely *S. aureus* (44.2%), MRSA (22.1%) and *E. faecium* (21.2%) [47]. Gram-positive *C. difficile* is recognised as the leading cause of HAI infective diarrhoea and community-acquired cases of colitis, promoted by its extensive drug resistance, spore-forming and toxin-producing capabilities [48]. *C. difficile* BSIs, however, are typically polymicrobial and associated with GNB, while *C. difficile* infections are associated with severe sepsis, with rates of ca. 70% [44]. Research has demonstrated that polymicrobial infection is a risk factor for severe sepsis [49]. Studies report that the incidence and mortality of Gram-positive sepsis are increasing, and Gram-negative sepsis is more severe where the host response and bacterial components impact on severity [3]. GNB from the ESKAPE category such as *E. coli* and *P. aeruginosa* are the leading causes of hospital-acquired BSIs, with greater than 33% mortality after 30 days [50]. For example, from 2020 to 2021, the mortality of HAI *P. aeruginosa* bacteraemia in the United Kingdom (UK) was ca. 34% compared to ca. 24% for community-acquired *P. aeruginosa* bacteraemia [51]. Studies report BSIs associated with *E. coli* (35.42%) resistant to aminoglycosides, cephalosporins, penicillin, fluoroquinolones, and B-lactam combination agents, *K. pneumonia* (19.74%) resistant to cephalosporins, fluoroquinolones, and trimethoprim/sulfamethoxazole, and the *Acinetobacter* species (9.67%) [52]. The LPA toxin released during BSIs with these pathogens varies amongst each GNB species, with its structure, length, lipid chain and sugar unit content determining its endotoxic pyrogenic effect [53]. Gram-negative *E. coli* and *K. pneumonia* are associated with ca. two-thirds of neonate sepsis cases in Ethiopia, where antibiotic resistance is a contributor to mortality [8]. HAI BSIs resultant from *Klebsiella* and *P. aeruginosa* increased between August 2020 and February 2021 in the UK and reached their highest levels since 2017 [54]. BSIs associated with MDR species vancomycin-resistant Enterococcus (VRE), beta-lactam-resistant *Klebsiella*, carbapenem-resistant *Acinetobacter* species are associated with increased risk of mortality [55]. Catheter-related BSIs increase mortality risk by 2.27 times [47]. Bacterial biofilms (communities of microbes attached to a biotic or abiotic surface) of *S. aureus*, *E. coli*, *K. pneumonia*, *P. aeruginosa*, *Proteus mirabilis* amongst other species on medical devices are commonly associated with HAIs. Biofilms of the Gram-positive species *S. aureus* and *S. epidermidis* are associated with prosthetic heart valve infections (ca. 50%), catheter infections (ca. 70%), and 87% of BSI infections [56]. Biofilms present on indwelling devices are extremely resistant to antibiotic therapy, disinfection protocols, and the host’s immune system [10], requiring a higher dose of therapeutics and removal of the medical device in chronic cases [57]. The early administration of antibiotic therapy is key to improving bacteraemia and sepsis prognosis in patients, with AMR having a negative impact on disease outcome [58]. For example, vancomycin or daptomycin is applied in the treatment of MRSA BSIs with ca. 50% of patients being non-responsive to therapy, allowing for persistent bacteraemia in the patient [59]. The identification of persistent cells and small colony variants (SCVs), both of which are slow-growing phenotypic variants having resistance to bactericidal antibiotics, also impacts treatment protocols [60].

### 3.2. Fungal Sepsis

Sepsis resulting from fungal pathogens is typically hospital acquired with invasive fungal infections (IFIs), having a mortality of ca. 60% in ICU patients [61]. The causative species of nosocomial IFIs include *Candida* spp., *Aspergillus* spp., and *Cryptococcus* spp. result in candidiasis, aspergillosis, and cryptococcal meningitis, respectively [62]. Data show that fungi are causative of ca. 20% of sepsis cases, with *Candida albicans* and *Candida glabrata* being most common, followed by the *Aspergillus* and *Cryptococcus* species [63]. The *Candida* species are associated with 93% of fungal nosocomial BSIs, are prevalent in late-onset sepsis aetiologies in neonates [64] and are associated with mortality rates of up to 70% [64]. Approximately 35% of Candidemia patients present with sepsis, or septic shock with 30% of cases in the ICU [65]. Disseminated infections and sepsis are also associated with *Aspergillus*, *Cryptococcus*, *Penicillin*, *Mucorales* and *Pneumocystis* amongst other species [66]. Risk factors for fungal infections and sepsis include treatment with empirical antibiotics, immunosuppressive drugs, and exposure to indwelling medical devices [44]. Additional clinical risk factors include neutropenia, hematopoietic stem cell or solid organ transplantation, and therapeutic high-dose corticosteroid [67]. The studies of Prout et al. (2019) show that paediatric patients having chronic morbidities such as congenital heart and hematologic disease, indwelling medical devices, and short bowel syndrome have an increased risk of fungal infections [68]. The risk of IFIs is also higher in very low birth weight infants and infants receiving parenteral nutrition [69]. Very low birth weights infants are immunocompromised and exposed to invasive medical devices, such as endotracheal tubes and central vascular catheters, increasing their risk of IFIs [70].

IFIs are currently diagnosed by mycological testing and blood cultures which have limitations including the slow growth rate of certain fungi [63]. Therapeutic treatment of fungal BSIs relies on drug classes polyenes, echinocandins, azoles and flucytosine where antifungal resistance is an issue [64]. More recently, the emergence of the critically important priority fungal pathogen MDR *Candida auris* in hospital settings represents a serious risk [71]. Studies report a high prevalence of *C. auris* in patients receiving mechanical ventilation, gastrostomy tubes, or urinary catheters with mortality rates of invasive *C. auris* reaching ca. 60% globally [72]. The Centre for Disease Control (CDC) recommends echinocandins as an antifungal therapy for *C. auris* infection; however, the emergence of resistance limits the therapeutic options [73]. Nosocomial *Aspergillus* are associated with mechanical ventilators leading to pulmonary aspergillosis with subacute invasive pulmonary aspergillosis (IPA) having high mortality rates [74] of 40 to 90% in immunocompromised patients [75]. Studies show that invasive aspergillosis is increasingly associated with chronic obstructive pulmonary disease (COPD), and COPD is an underlying morbidity in 34% of ICU invasive aspergillosis patients, with cancer and organ transplantation associated risk factors [76]. Importantly, studies show a mortality rate of ca. 70% from invasive aspergillosis [76]. Furthermore, other studies show a mortality rate of ca. 32% in IPA patients 12 months post diagnosis [77]. Antifungal agents have biocompatibility issues with therapeutic failure also associated with drug properties (pharmacokinetic and pharmacodynamic) and drug–drug interactions in patients [78]. Additionally, MDR is common in fungal pathogens including the clinical *Candida* species, *Cryptococcus*, and *Aspergillus* [78]. Azole resistance is also emerging in *Aspergillus* species including *A. fumigatus* [79]. The broad application of agricultural fungicides has contributed to the emergence of therapeutic resistance in species such as *C. auris* [80]. Similarly, invasive or disseminated Cryptococcal infections are associated with immunocompromised patients, particularly HIV patients, cancer patients and pulmonary cryptococcosis [28]. The main species involved in IFIs are *C. neoformans* and *C. gattii*; however, the cases are primarily community acquired via inhalation of fungal spores present in the environment [81].

## 4. Sepsis Detection towards Early Diagnosis

Empiric therapy is impacted by antimicrobial stewardship in an era of antimicrobial resistance and emerging and re-emerging pathogens. As clinical pathogens become increasingly difficult to treat, early detection and disease prevention become increasingly important. Making therapeutic decisions in sepsis treatment is often not straightforward as clinical signs of sepsis vary and are nonspecific, particularly in immunocompromised patients where blood culturing is frequently associated with false negatives, requires ca. 72 h, and lacks sensitivity [82]. Importantly, ca. 50% of cultured cases of sepsis are determined to be culture negative [83]. To successfully prevent cases of sepsis and associated mortality, early detection and the timely application of appropriate therapeutics is essential [67]. Evidence shows an 8% increase in mortality risk in sepsis shock patients for every hour delay in treatment [84].

### 4.1. Biomarkers for Sepsis Diagnosis and Disease Progression

The application of biomarkers for early detection of infectious diseases allows for improved therapeutic stewardship. A biological biomarker is a measurable indicator (ideally accurate and reproducible) of disease status in a patient which is absent or reduced in healthy persons, and which can also indicate a response to therapy [85]. Sepsis triggers several biochemical and immune inflammatory pathways, releasing numerous mediators which could act as biomarkers such as acute-phase proteins, erythrocyte sedimentation rate, pro-inflammatory cytokines, chemokines, DAMPs, endothelial cell markers, and leukocyte surface markers, amongst others [86] (Table 2). Measurements of the C-reactive protein (CRP) and procalcitonin (PCT) serve as biomarkers in the treatment of infectious disease [87]. Both, however, are non-specific markers of inflammation and associated with non-infectious inflammatory morbidities. The pro-inflammatory cytokine IL-6 has potential as a biomarker in infectious disease as it increases earlier than PCT and CRP, potentially allowing for early detection [88]. The research of Shi et al. (2024) determined that PCT has improved efficacy over CRP in distinguishing between Gram-positive and Gram-negative sepsis, particularly with *E. coli* as a causative agent [89]. The TLR presepsin is elevated in the early stages of sepsis and is specific for infectious disease, with levels increasing within 2 h [90]. The triggering receptor expressed on myeloid cells-1 (TREM-1) is a transmembrane receptor present on innate immune cells, platelets, and endothelial cells, which is released as a soluble factor termed sTREM-1 during incidence of disease, including septic shock [91]. High sTREM-1 levels are present in critical sepsis ICU patients in the early stages of septic shock, suggesting the potential of sTREM-1 as a suitable biomarker [91]. Studies suggest that assessing presepsin and CRP, PCT or IL-6 together may improve diagnostic procedures and patient outcomes particularly in neonatal sepsis [92]. The application of a panel of seven biomarkers identified 89% of VAP patients and 100% of non-VAP patients, with a similar combination of biomarkers having increased efficacy compared to PCT alone [86]. Pathogen-specific biomarkers, such as direct antigen tests are important in diagnosing cases of morbidity [83]. Antigen tests are available for many sepsis-associated pathogens, including *Candida* (detecting 1,3)-β-D-glucan (BDG), *C. difficile* (glutamate dehydrogenase (GDH)) and other species; however, their sensitivity towards sepsis is limited [83]. The chromogenic limulus amoebocyte lysate assay was the first diagnostic test developed to detect LPS toxin of GNB but lacks specificity, with the chemiluminescent Endotoxin Activity Assay (EAA) having much improved sensitivity and specificity [93].

At present, no biomarker has displayed sufficient accuracy to detect sepsis as distinct from infectious disease without sepsis [82]. The application of these biomarkers is difficult in neonatal sepsis due to non-specific concentrations and an absence of cutoff levels for each biomarker [92]. The absence of cutoff values or use of a broad range of values, differences in the classification of sepsis and lack of standardised analytical methods are major hurdles in the application of biomarkers in diagnosing sepsis in patients. Sepsis is a heterogenous condition with varying pathophysiology between patients impacting immune responses with patient factors such as age, sex, infection type, co-morbidities and epigenetics contributing to sepsis heterogenicity. More specific, accurate and reproducible biomarkers for sepsis need to be investigated to identify important biological components and cutoff values corresponding to their clinical significance, disease progression and response to therapy. Indeed, the application of a multi-biomarker approach may prove necessary in establishing a more accurate picture of sepsis aetiology at a cellular level.

### 4.2. Omics Technology—Machine Learning and Microfluidics

Omics technology includes the application of high-throughput biochemical assays based on cytomics, genomics, transcriptomics, proteomics, epigenomics, and metabolomics in the study of disease pathogenesis. The assessment of cellular responses, nucleic acid, protein activity, metabolite levels, and external factors in the pathogenesis of sepsis may provide novel accurate biomarkers for disease onset and progression. The studies of Mickiewicz et al. (2018) described the suitability of metabolomics in the triage of infant sepsis patients [97]. The application of a combined omics analysis may provide a more detailed pathology of systemic response and local tissues responses in sepsis cases. The studies of Li et al. (2023) applied an integrative analysis of multi-omics to characterise changes in sepsis pathogenesis and identify molecular markers for sepsis treatment [98]. Additional research applied proteomics and metabolomics to assess the exhaustion of antioxidant defences and oxidative stress and the comprehensive impairment of the mitochondria in skeletal muscle of sepsis patients [13]. Meta-analyses of the data generated from omics allow for data pattern analysis and the categorising of clinical data according to sepsis type and severity into endotypes, which may improve patient outcomes. Removing bias and the influence of confounding variables remain a challenge in a clinical setting. The application of a multi-omics approach may allow for patient-specific biomarkers based on gene and protein expression of cells in combination with clinical features. Additional biomarkers under investigation include cell-free DNA and monocyte activation, and microbiomics [99].

Machine learning, which applies mathematical methods to large datasets, allows for the development of sepsis prediction algorithms [100]. Studies have shown that the application of machine learning algorithms has successfully predicted sepsis, reduced hospital stay durations, and decreased mortality by ca. 12% [101]. The application of machine learning by Alanazi et al. (2023) predicted sepsis in ICU patients with better sensitivity than traditional models [102]. Similar studies concluded that machine learning can act as a predictive tool for sepsis mortality and early risk endotyping compared to current clinical scoring systems [11]. The Duke Institute for Health Innovation developed a machine learning model, Sepsis Watch^TM^, as an early warning system for sepsis risk in clinical settings [103]. Sepsis Watch^TM^ outperformed current scoring methods for sepsis and detected sepsis 5 h quicker in clinical settings [104]. Studies applied machine learning to identify patients displaying certain sepsis phenotypes and responding to recombinant human thrombomodulin (rhTM) therapy [105]. A Targeted Real-time Early Warning System (TREWS) for sepsis has been designed and investigated to improve patient outcomes [106]. In silico models incorporating omics with additional information from clinical parameters and different sources may allow for improved drug treatment of sepsis as part of drug discovery and repurposing [107]. To achieve sensitivity and accuracy with machine learning, the selection of relevant variables and combinations such as lactic acid is essential to predict sepsis onset and patient outcomes. The combination of machine learning with artificial intelligence (AI) for the detection of sepsis has undoubtedly gained much attention. At present, however, there are no regulatory frameworks designed for learning systems, with an absence of high-quality clinical trials to determine the impact on patient outcomes.

In vitro methods including organ-on-chip may offer insight into sepsis management and allow for improved therapeutic screening [108]. Organ-on-chip or 3-D biomimetic microfluidic assays are a combination of microfluidic and tissue engineering, designed to synthesize the cell organization and physical parameters of an organ [109]. A microfluidic LabDisk technology developed as part of the ASCMicroPlat project aims to develop new test methods for rapid diagnosis of neonatal sepsis. LabDisk, incorporating different biomarkers and assays, showed sensitivity and specificity to enable the detection of low bacterial loads and prevent non-specific signal generation in clinically relevant samples [110]. SeptiCyte RAPID, an mRNA test to identify sepsis from non-infectious systemic inflammation using reverse transcription polymerase chain reaction (PCR) to quantify the relative expression levels of host response genes in inflammatory patients, has been approved by the US Food and Drug Administration (FDA) [111]. The use of microfluidic platforms and multiplex technology offers many advantages in the diagnosis of infectious disease, including rapid operation times, low reagent volumes, high integration capability, and improved sensitivity and specificity [112]. Excellent reviews on the application of microfluidics are provided [11,100,113].

## 5. Antimicrobial Peptides and Phages as Novel Therapeutic Options

Antimicrobial peptides (AMPs) have demonstrated antibacterial efficacy against numerous AMR and MDR pathogens. AMPs are components of the biological innate immune system having antimicrobial activity including anti-biofilm and sporicidal action, anti-inflammatory, anti-cancer, and tissue regeneration properties [114]. AMPs achieve immune modulatory activity by recruiting and activating immune cells and altering TLR recognition of microbial species [115]. As an antibacterial agent, the action of AMPs is a multi-hit approach with cell lysis from membrane damage, a major cause of cell death [9]. Certain AMPs also have an ability to bind and inhibit bacterial endotoxins, conferring an anti-virulence action on the AMP. Such activity coupled with pro- and anti-inflammatory immune modulation, may aid in the control of sepsis in vivo. Bacitracin has demonstrated a neutralising action against the exotoxins of *C. botulinum*, *C. perfringens* and *C. difficile* and *B. anthracis* lethal toxin [116]. Defensins are AMPs produced by plant and animal species that have a role in innate immunity and maintaining the microbiota balance of the host. Defensins have an anti-toxin action by binding to and unfolding bacterial toxins and thus increasing their susceptibility to proteolysis [117]. The human defensins HNP and HD5 inhibit the *B. anthracis* toxin, diphtheria toxin, *P. aeruginosa* exotoxin, and *C. difficile* cytotoxin B [118]. The mammalian cathelicidin AMPs LL-37 LL-32, CAP18, CRAMP, BMAP-27/28 have displayed LPS-neutralising activity [119]. LL-37 has immune modulation activity by regulating immune mediators, including pro-inflammatory IL-10, IL-8 and type 1 interferons, and the release of inflammatory mediators, LL-37. The anti-inflammatory action inhibits the inflammasomes, TNF-alpha, and IL-12 [120]. LL-37 is the only cathelicidin AMP currently known to be present in humans and to directly neutralise LPS in vivo [121]. Studies demonstrate that LL-37 reduced vascular nitric oxide production, following LPS exposure, by neutralising the LPS toxin [122]. In vivo studies demonstrated that LL-37 increased the survival of septic mice by protecting the macrophages, inducing pro-inflammatory cytokines, and stimulating neutrophil production [123]. LL-37 has chemotactic activity towards the immune cells including neutrophils, monocytes, mast cells, and T cells and influences the expression of the genes associated with chemokines and their receptors [124]. Furthermore, LL-37 and its analogues have demonstrated activity against *C. albicans*, *C. tropicalis*, *C. krusei*, *C. parapsilosis* in vitro [125]. At present, colistin and gramicidin are two AMPs implemented as last resort antibacterial therapeutics [114]. Colistin is used to treat MDR Gram-negative bacteria, including *P. aeruginosa*, *A. baumannii*, and *K. pneumoniae* [126]. Studies describe increased survival rates in neonatal patients having sepsis from MDR GNB species treated with colistin [127]. Broad spectrum application of AMPs is hindered by their biocompatibility issues, sensitivity to proteases in vivo, and large-scale production issues (Table 3) [128]. Colistin is associated with nephrotoxicity and neurotoxicity [127]. Colistin is associated with acute kidney injury in ca. 53% of patients [126]. Alarmingly, resistance to colistin has been identified in *K. pneumonia*, with such species having a high mortality rate [47]. Gramicidin is not used in vivo due to its hemolytic side-effects but is formulated for dermal application [129]. Genetic engineering, post-translational modification of AMPs, optimisation of biological expression systems for large-scale production and prodrug formulation may overcome some production and application hurdles [130]. Designing AMPs to have the correct ratio of hydrophobicity to hydrophilicity may ensure antimicrobial activity with reduced cytotoxicity.

Bacteriophages (phages) are viruses which selectively and specifically infect bacterial cells resulting in cell death upon lysis of the host cell. Phages have demonstrated efficacy against MDR species including nosocomial relevant *S. aureus*, *E. faecium*, with mycoviruses which selectively infect fungal species thus demonstrating efficacy against the *A. fumigatus* and *Candida* species [131]. The FDA has approved phage treatment for illnesses including infections of prosthetic joints, bone and implant infections, wound infections, diabetic foot infections, and acute tonsillitis [9]. The studies of Kaabi et al. (2020) demonstrated the efficacy of a phage cocktail against neonatal sepsis isolates including ESKAPE pathogens *E. coli*, *K. pneumonia* and *P. aeruginosa* [132]. Studies investigating the use of a phage cocktail in a mice model concluded that low repeat dosages with the monitoring of vital signs may aid in the treatment of septicaemia associated with MDR Gram-negative bacteria, including MDR *K. pneumonia* [133]. Similarly, studies demonstrated the efficacy of phage therapy against *A. baumannii* sepsis in mice models [134]. The M13 phage also demonstrated efficacy in sepsis mice models and reduced the level of LPS-induced inflammatory responses [135]. Phage treatment of mice models having bacteraemia had a survival rate of 100% with no pathogens isolated 96 h post treatment [136]. A phage cocktail administered to a septic acute kidney injury patient for ca. 10 days was associated with a negative bacteria count, reduced CRP, and recovery of renal function in the patient [137]. The release of LPS from the Gram-negative outer membrane following phage cell lysis is a risk associated with phage application. Studies have reported LPS release in the phage treatment of MDR *P. aeruginosa*, with an inflammatory cytokine reaction established [138]. Studies are warranted on determining the impact of such LPS release on immune mediators and the potential for triggering a cytokine storm. Similar to AMPs, phages have large scale production, formulation, stability and pharmacokinetic issues which must be overcome before systemic application can be achieved [114].

**Table 3:** Advantages and limitations associated with the use of AMPs and Phages in the treatment of sepsis.

Potential Novel Therapeutic	Advantages	Limitations
Antimicrobial peptides	Modulation of immune responses, pro- and anti-inflammatory	In vivo efficacy not fully established
	Potent broad-spectrum efficacy against MDR species	Limited pharmacokinetic profiling coupled with biocompatibility issues [127]
	Resistance to AMPs is not common	Large-scale synthesis is costly
	Can be used in synergy to antibiotics	Downstream processing and formulation considerations
	Cathelicidin AMPs, e.g., LL-37 have LPS neutralising action [119]	Half-life, stability, and enzymatic degradation in vivo [9]
	Amenable to post-translational modification and genetic engineering [114]	Limited in vivo studies on sepsis control
	Promote proteolysis of bacterial toxins [117]	Binding to serum proteins in vivo may hinder bioavailability [114]
	LL-37 influences chemokine gene expression [124]	LL-37 results in haemolytic damage at MIC range [9]
Phage’s	Potent, selective, and specifically target species [132]	Large-scale production and formulation issues [9]
	Self-limiting once pathogen is cleared	Bacterial resistance may develop [114]
	Can be applied as a phage cocktail [133]	Immune system clearance of phages may reduce activity in vivo [9]
	Effective against MDR species [133]	Stability and storage issues [9]
	Biocompatible for patient use [9,132]	Phage may transmit AMR genes [131]
	May regulate inflammatory responses in vivo [135]	Phage may transmit genes coding for toxins [9]
	Limited impact on patient microbiota	Risk of cytokine storm in patient; needs investigating [138]

## 6. Conclusions

Sepsis is resultant from a dysregulation of the immune system following the exposure to an infectious pathogen which may be bacterial, fungal, or viral. Microbial cell components trigger a dysregulation of the immune response in the patient which results in organ failure and mortality. Gram-negative bacterial species are particularly problematic due to the release of the LPS toxin upon cell death. Mortality rates of nosocomial transmitted sepsis are high, especially in ICU and neonate patients. With the increasing prevalence of immunocompromised patients, patients having co-morbidities and the proliferation of antimicrobial resistance, empiric therapeutic intervention has become problematic in clinical settings. Furthermore, ca. 75% of sepsis recovery patients have higher rates of long-term morbidities including mental health issues, cognitive difficulties, physical issues, and mortalities post discharge, i.e., post-sepsis syndrome. Early detection of sepsis or septic shock aids in sepsis triage and guiding optimal therapeutic administration. Inflammatory biomarkers such as CRP and PCT do not provide specific information of disease aetiology and differentiation between infectious and non-infectious immune responses. Novel specific markers need to be identified and assessed in sepsis cases, such as sTREM-1 and presepsin. Additionally, cutoff values and dose–response relationships need to be established to allow for the monitoring of disease progression and response to therapy. The application of machine learning and AI allows for the development of sepsis algorithms which can support clinical decisions in sepsis management. Advances in organ-on-chip technology also show promise in establishing better diagnostic and treatment interventions. The application of AMPs and bacteriophages in the treatment of infectious disease may offer novel therapeutic approaches as standalone or adjuvant therapies. Indeed, certain AMPs demonstrate antibacterial, anti-virulence, and immune modulation activity. The AMP LL-37 was found to directly neutralise LPS in vivo. As progress is being made in understanding the aetiology of sepsis, improved diagnosis has been achieved. There is much research needed, however, to identify sensitive and specific biomarkers and novel therapeutics to safeguard at-risk groups in clinical settings. The identification of such biomarkers may also allow for a more definite uniform definition of sepsis to be applied clinically.

## Figures and Tables

**Table 2:** Biomarkers having potential for use in the diagnosis of infectious sepsis.

Biomarker	Description	Sepsis Concentrations—Time of Onset (Hours)	Limitations
C-Reactive Protein (CRP)	Acute phase protein	>50 μg/mL—ca. 6 h	Low specificity as increases in many inflammatory diseases, production varies in neonates e.g., preterm [92]
Serum Amyloid A	Acute-phase protein secreted by liver and adipose cells	>1 mg/mL—ca. 24 h	Studies on levels excreted by neonates needed [94]
Procalcitonin (PCT)	Precursor of calcitonin produced by C-cells of the thyroid gland [88]	>2 ng/mL—12–24 h	Cannot be used independently [95]
IL-6	Pro-inflammatory cytokine–interleukin [88]	>1000 pg/mL—6 h	Half-life of 1 h, Inferior to PCT and CRP [95]
Presepsin (sCD14-ST)	Soluble CD14, Toll-like receptor (TLR) [90]	>400–600 pg/ml—2 h [90]	Levels also increase during liver cirrhosis, diabetes mellitus and heart failure, leads to non-specificity [92]
CD 64	CD64 is a high-affinity immunoglobulin Fc γ receptor expressed on monocytes, eosinophils, and neutrophils,	>800 mcL—1–6 h	More suitable for early stages of sepsis diagnosis
Soluble TREM-1 (sTREM-1) [91]	Triggering receptor expressed on myeloid clles-1	400 pg/mL—2–6 h [91]	Moderate ability in diagnosing sepsis, rapid sTREM-1 clearance, and short half-life in vivo [91]
Adrenomedullin (ADM) and Pro adrenomedullin (proADM) [95]	ADM and proADM is a stable and detectable fragment of 48-amino acids produced by vascular endothelial cells and smooth muscle cells [96]	ADM ADM was 74 pg/mL in sepsis patients, 107 pg/mL in septic shock, and 29 pg/mL in non-septic cases—<24 h [85] proADM 1.4 nmol/L [96]	ADM has short half-life and rapid clearance, difficult to measure in clinical setting [96]
Pathogen-specific biomarkers	Antigen tests, immunoassays	Limited sensitivity for sepsis in absence of pathogens [83]	

## References

[1] Singer M., Deutschman C.S., Seymour C.W., Shankar-Hari M., Annane D., Bauer M., Bellomo R., Bernard G.R., Chiche J.D., Coopersmith C.M. (2016). The Third International Consensus Definitions for Sepsis and Septic Shock (Sepsis-3). JAMA.

[2] Levy M.M., Evans L.E., Rhodes A. (2018). The Surviving Sepsis Campaign Bundle: 2018 update. Intensive Care Med..

[3] Tang A., Shi Y., Dong Q. (2023). Prognostic differences in sepsis caused by gram-negative bacteria and gram-positive bacteria: A systematic review and meta-analysis. Crit. Care.

[4] Li J., Liu H., Wang N. (2024). Persistent high sepsis-induced coagulopathy and sequential organ failure assessment scores can predict the 28-day mortality of patients with sepsis: A prospective study. BMC Infect. Dis..

[5] Sartelli M., Kluger Y., Ansaloni L., Hardcastle T.C., Rello J., Watkins R.R., Bassetti M., Giamarellou E., Coccolini F., Abu-Zidan F.M. (2018). Raising concerns about the Sepsis-3 definitions. World J. Emerg. Surg..

[6] Fleischmann-Struzek C., Mellhammar L., Rose N. (2020). Incidence and mortality of hospital- and ICU-treated sepsis: Results from an updated and expanded systematic review and meta-analysis. Intensive Care Med..

[7] Lei S., Li X., Zhao H., Xie Y., Li J. (2022). Prevalence of sepsis among adults in China: A systematic review and meta-analysis. Front. Public Health.

[8] Solomon S., Akeju O., Odumade O.A., Ambachew R., Gebreyohannes Z., Van Wickle K. (2021). Prevalence and risk factors for antimicrobial resistance among newborns with gram-negative sepsis. PLoS ONE.

[9] Garvey M. (2024). Medical Device-Associated Healthcare Infections: Sterilization and the Potential of Novel Biological Approaches to Ensure Patient Safety. Int. J. Mol. Sci..

[10] Meade E., Savage M., Garvey M. (2021). Effective Antimicrobial Solutions for Eradicating Multi-Resistant and β-Lactamase-Producing Nosocomial Gram-Negative Pathogens. Antibiotics.

[11] Zhang Y., Xu W., Yang P. (2023). Machine learning for the prediction of sepsis-related death: A systematic review and meta-analysis. BMC Med. Inform. Decis. Mak..

[12] Dolin H.H., Papadimos T.J., Chen X., Pan Z.K. (2019). Characterization of Pathogenic Sepsis Etiologies and Patient Profiles: A Novel Approach to Triage and Treatment. Microbiol. Insights.

[13] Duceau B., Blatzer M., Bardon J. (2022). Using a multiomics approach to unravel a septic shock specific signature in skeletal muscle. Sci. Rep..

[14] Bullock B., Benham M.D. (2024). Bacterial Sepsis. [Updated 2023 May 21]. StatPearls [Internet].

[15] van der Slikke E.C., Beumeler L.F.E., Holmqvist M., Linder A., Mankowski R.T., Bouma H.R. (2023). Understanding Post-Sepsis Syndrome: How Can Clinicians Help?. Infect. Drug Resist..

[16] Dahlberg J., Linder A., Mellhammar L. (2023). Use of healthcare before and after sepsis in Sweden: A case-control study. BMJ Open.

[17] Rocheteau P., Chatre L., Briand D. (2015). Sepsis induces long-term metabolic and mitochondrial muscle stem cell dysfunction amenable by mesenchymal stem cell therapy. Nat. Commun..

[18] Buchman T.G., Simpson S.Q., Sciarretta K.L., Finne K.P., Sowers N., Collier M. (2020). Sepsis among medicare beneficiaries: 1. The burdens of sepsis, 2012–2018. Crit. Care Med..

[19] Srzić I., Nesek A.V., Tunjić P.D. (2022). Sepsis Definition: What’s New  in the Treatment Guidelines. Acta Clin. Croat..

[20] (2024). Horizon. https://projects.research-and-innovation.ec.europa.eu/en/horizon-magazine/deadly-sepsis-and-antibiotic-resistant-bacteria-are-europes-crosshairs.

[21] Kim J.H., Kordahi M.C., Chac D., DePaolo W. (2019). Toll-like receptor-6 signaling prevents inflammation and impacts composition of the microbiota during inflammation-induced colorectal cancer. Cancer Prev. Res..

[22] Wiersinga W.J., van der Poll T. (2022). Immunopathophysiology of human sepsis. eBioMedicine.

[23] He Y., Peng Y., Tao L., Peng Z., Yang H. (2020). Peroxiredoxin-1 aggravates lipopolysaccharide-induced septic shock via promoting inflammation. Biochem. Biophys. Res. Commun..

[24] Zhou M., Aziz M., Wang P. (2021). Damage-associated molecular patterns as double-edged swords in sepsis. Antioxid. Redox Signal..

[25] Lu M., Ma A., Liu J. (2022). Study on the expression of TRIM7 in peripheral blood mononuclear cells of patients with sepsis and its early diagnostic value. BMC Infect. Dis..

[26] Hellenthal K.E.M., Brabenec L., Wagner N.M. (2022). Regulation and Dysregulation of Endothelial Permeability during Systemic Inflammation. Cells.

[27] Xin L., Mubing Q., Harold J., Yanxia G., Shiyuan Y., Zengzheng G., Chao G., Huadong Z., Djillali A., Yi L. (2023). Clinical Phenotypes of Sepsis-Associated Encephalopathy: A Retrospective Cohort Study. Shock.

[28] Huang M., Cai S., Su J. (2019). The Pathogenesis of Sepsis and Potential Therapeutic Targets. Int. J. Mol. Sci..

[29] Cui J., Oehrl S., Ahmad F., Brenner T., Uhle F., Nusshag C., Rupp C., Funck F., Meisel S., Weigand M.A. (2021). Detection of In Vivo Inflammasome Activation for Predicting Sepsis Mortality. Front. Immunol..

[30] Deng M., Tang Y., Li W., Wang X., Zhang R., Zhang X., Zhao X., Liu J., Tang C., Liu Z. (2018). The Endotoxin Delivery Protein HMGB1 Mediates Caspase-11-Dependent Lethality in Sepsis. Immunity.

[31] Wang Y.C., Liu Q.X., Liu T., Xu X.E., Gao W., Bai X.J. (2018). Caspase-1-dependent pyroptosis of peripheral blood mononuclear cells predicts the development of sepsis in severe trauma patients: A prospective observational study. Medicine.

[32] Anderson U., Yang H. (2022). HMGB1 is a critical molecule in the pathogenesis of Gram-negative sepsis. J. Intensive Med..

[33] Garvey M. (2023). The Association between Dysbiosis and Neurological Conditions Often Manifesting with Chronic Pain. Biomedicines.

[34] Prescott H.C., Ostermann M. (2023). What is new and different in the 2021 Surviving Sepsis Campaign guidelines. Med. Klin. Intensivmed. Notfmed..

[35] Taniguchi L.U., Pires E.M.C., Vieira J.M., Azevedo L.C.P. (2017). Systemic inflammatory response syndrome criteria and the prediction of hospital mortality in critically ill patients: A retrospective cohort study. Rev. Bras. Ter. Intensiva..

[36] Chakraborty R.K., Burns B. (2024). Systemic Inflammatory Response Syndrome. StatPearls [Internet].

[37] Kaukonen K.M., Bailey M., Suzuki S., Pilcher D., Bellomo R. (2014). Mortality Related to Severe Sepsis and Septic Shock Among Critically Ill Patients in Australia and New Zealand, 2000–2012. JAMA.

[38] Bauer M., Gerlach H., Vogelmann T. (2020). Mortality in sepsis and septic shock in Europe, North America and Australia between 2009 and 2019—results from a systematic review and meta-analysis. Crit. Care.

[39] Zhao P.Y., Xia Y., Tao Z.B., Li S.Y., Mao Z., Yang X.P., Yao R.Q., Du X.H. (2022). Global Research Status of Multiple Organ Dysfunction Syndrome During 2001–2021: A 20-Year Bibliometric Analysis. Front. Med..

[40] Westphal G.A., Pereira A.B., Fachin S.M., Barreto A.C.C., Bornschein A.C.G.J., Caldeira F.M., Koenig Á. (2019). Characteristics and outcomes of patients with community-acquired and hospital-acquired sepsis. Rev. Bras. Ter. Intensiva..

[41] Markwart R., Saito H., Harder T. (2020). Epidemiology and burden of sepsis acquired in hospitals and intensive care units: A systematic review and meta-analysis. Intensive Care Med..

[42] Ahiawodzi P.D., Okafor I., Chandler S., Kelly K., Thompson D.K. (2020). Indwelling medical device use and sepsis risk at a health professional shortage area hospital: Possible interaction with length of hospitalization. Am. J. Infect. Control.

[43] Wang L., Li D., Chen Z., He L., Wang X., Tao L. (2022). An Atypical Case of Monomicrobial Clostridioides difficile Septicemia with No Gastrointestinal Manifestations. Front. Cell. Infect. Microbiol..

[44] Garvey M., Rowan N.J. (2023). Pathogenic Drug Resistant Fungi: A Review of Mitigation Strategies. Int. J. Mol. Sci..

[45] Pickens C.I., Wunderink R.G. (2022). Methicillin-Resistant Staphylococcus aureus Hospital-Acquired Pneumonia/Ventilator-Associated Pneumonia. Semin. Respir. Crit. Care Med..

[46] Denis J.B., Lehingue S., Pauly V., Cassir N., Gainnier M., Léone M., Daviet F., Coiffard B., Baron S., Guervilly C. (2019). Multidrug-resistant Pseudomonas aeruginosa and mortality in mechanically ventilated ICU patients. Am. J. Infect. Control..

[47] Singh N., Puri S., Anshul K.S., Pahuja H., Kalia R., Arora R. (2023). Risk Factors and Outcome Analysis of Gram-Positive Bacteremia in Critically Ill Patients. Cureus.

[48] Garvey M. (2023). Foodborne Clostridioides Species: Pathogenicity, Virulence and Biocontrol Options. Microorganisms.

[49] Montull B., Menéndez R., Torres A., Reyes S., Méndez R., Zalacaín R. (2016). Predictors of severe sepsis among patients hospitalized for community-acquired pneumonia. PLoS ONE.

[50] Choy B., Krutikov M., El-Mugamar H., Paget S., Hsu D., Sivaramakrishnan A. (2023). Hospital onset, healthcare assoicataed Gram negative bloodstream infections in patients admitted to a busy district general hospital in England: A retrospective cohort study. J. Hosp. Infect..

[51] UK HSA (2021). UK Health Security Agency. Thirty-Day All-Cause Mortality Following MRSA, MSSA and Gram-Negative Bacteraemia and C. Difficile Infections, 2020 to 2021.

[52] Juliana A., Leela K.V., Gopinathan A., Sujith R. (2022). Occurrence and Resistance Pattern of Gram-Negative Bacteremia and Sepsis in A Tertiary Care Hospital-A Four-Year Study. J. Pure Appl. Microbiol..

[53] Fux A.C., Casonato Melo C., Michelini S., Swartzwelter B.J., Neusch A., Italiani P., Himly M. (2023). Heterogeneity of Lipopolysaccharide as Source of Variability in Bioassays and LPS-Binding Proteins as Remedy. Int. J. Mol. Sci..

[54] Sloot R., Nsonwu O., Chudasama D., Gerver S., Brown C., Hope R., Mason E., Springer A. (2021). Rising rates of hospital onset Klebsiella spp. and Pseudomonas aeruginosa bacteraemia in NHS acute trusts in England: A review of national surveillance data, August 2020-February 2021. J. Hosp. Infect..

[55] Vincent J.L., Sakr Y., Singer M., Martin-Loeches I., Machado F.R., Marshall J.C. (2020). Prevalence and outcomes of infection among patients in intensive care units in 2017. JAMA.

[56] Khatoon Z., McTiernan C.D., Suuronen E.J., Mah T.F., Alarcon E.I. (2018). Bacterial biofilm formation on implantable devices and approaches to its treatment and prevention. Heliyon.

[57] Sousa V., Mardas N., Spratt D., Hassan I.A., Walters N.J., Beltrán V., Donos N. (2022). The effect of microcosm biofilm decontamination on surface topography, chemistry, and biocompatibility dynamics of implant titanium surfaces. Int. J. Mol. Sci..

[58] Bassetti M., Rello J., Blasi F., Goossens H., Sotgiu G., Tavoschi L. (2020). Systematic review of the impact of appropriate versus inappropriate initial antibiotic therapy on outcomes of patients with severe bacterial infections. Int. J. Antimicrob. Agents.

[59] Adamu Y., Puig-Asensio M., Dabo B., Schweizer M.L. (2024). Comparative effectiveness of daptomycin versus vancomycin among patients with methicillin-resistant *Staphylococcus aureus* (MRSA) bloodstream infections: A systematic literature review and meta-analysis. PLoS ONE.

[60] Mikkaichi T., Yeaman M.R., Hoffmann A., MRSA Systems Immunobiology Group (2019). Identifying determinants of persistent MRSA bacteremia using mathematical modeling. PLoS Comput. Biol..

[61] Niederman M.S., Baron R.M., Bouadma L. (2021). Initial antimicrobial management of sepsis. Crit. Care.

[62] Rathore S.S., Sathiyamoorthy J., Lalitha C., Ramakrishnan J. (2022). A holistic review on Cryptococcus neoformans. Microb. Pathog..

[63] Lippi G., Cervellin G. (2019). Can presepsin be used for screening invasive fungal infections?. Ann. Transl. Med..

[64] Kotey F.C., Dayie N.T., Tetteh-Uarcoo P.B., Donkor E.S. (2021). Candida Bloodstream Infections: Changes in Epidemiology and Increase in Drug Resistance. Infect. Dis..

[65] Pfaller M.A., Carvalhaes C.G., Smith C.J., Diekema D.J., Castanheira M. (2020). Bacterial and fungal pathogens isolated from patients with bloodstream infection: Frequency of occurrence and antimicrobial susceptibility patterns from the SENTRY Antimicrobial Surveillance Program (2012–2017). Diagn. Microbiol. Infect. Dis..

[66] Prohaska S., Henn P., Wenz S. (2020). A case report of fatal disseminated fungal sepsis in a patient with ARDS and extracorporeal membrane oxygenation. BMC Anesthesiol..

[67] Evans L., Rhodes A., Alhazzani W., Antonelli M., Coopersmith C.M., French C. (2021). Surviving sepsis campaign: International guidelines for management of sepsis and septic shock 2021. Crit. Care Med..

[68] Prout A.J., Talisa V.B., Carcillo J.A., Decker B.K., Yende S. (2019). Bacterial and Fungal Etiology of Sepsis in Children in the United States. Crit. Care Med..

[69] Sanni U.A., Lawal T.O., Na’uzo A.M., Audu L.I. (2023). Invasive Fungal Infection Presenting as Early-Onset Neonatal Sepsis: A Case Report from Northern Nigeria. J. Clin. Neonatol..

[70] Gupta M., Mishra A.K., Singh S.K. (2018). *Cryptococcus laurentii* fungemia in a low birth weight preterm neonate: India. J. Infect. Public Health.

[71] Snyder G.M., Wright S.B. (2019). The epidemiology and prevention of Candida auris. Curr. Infect. Dis. Rep..

[72] Arensman K., Miller J.L., Chiang A., Mai N., Levato J., LaChance E., Anderson M., Beganovic M., Dela Pena J. (2020). Clinical Outcomes of Patients Treated for Candida auris Infections in a Multisite Health System, Illinois, USA. Emerg. Infect. Dis..

[73] (2024). CDC. https://www.cdc.gov/fungal/candida-auris/c-auris-health-qa.html#:~:text=Consultation%20with%20an%20infectious%20disease,and%20Control%20of%20Candida%20auris.

[74] Briones-Claudett K.H., Briones-Claudett M.H., Murillo V.R.A. (2020). Invasive Subacute Pulmonary Aspergillosis After Puerperal Sepsis: Case Report. J. Investig. Med. High Impact Case Rep..

[75] Tong X., Liu T., Jiang K., Wang D., Liu S., Wang Y., Fan H. (2021). Clinical Characteristics and Prognostic Risk Factors of Patients With Proven Invasive Pulmonary Aspergillosis: A Single-Institution Retrospective Study. Front. Med..

[76] Mir T., Uddin M., Khalil A., Lohia P., Porter L., Regmi N., Weinberger J., Koul P.A., Soubani A.O. (2022). Mortality outcomes associated with invasive aspergillosis among acute exacerbation of chronic obstructive pulmonary disease patient population. Respir. Med..

[77] Henao-Martínez A.F., Corbisiero M.F., Salter I., Chastain D.B., Thompson G.R. (2023). Invasive pulmonary aspergillosis real-world outcomes: Clinical features and risk factors associated with increased mortality. Med. Mycol..

[78] Garvey M., Meade E., Rowan N.J. (2022). Effectiveness of front line and emerging fungal disease prevention and control interventions and opportunities to address appropriate eco-sustainable solutions. Sci. Total Environ..

[79] Wiederhold N.P. (2021). Emerging Fungal Infections: New Species, New Names, and Antifungal Resistance. Clin. Chem..

[80] Casadevall A., Kontoyiannis D.P., Robert V. (2019). On the emergence of Candida auris: Climate change, azoles, swamps, and birds. mBio.

[81] Pan W.G., Chen B.C., Li Y.F. (2021). An unusual case of reactivated latent pulmonary cryptococcal infection in a patient after short-term steroid and azathioprine therapy: A case report. BMC Pulm. Med..

[82] Hung S.K., Lan H.M., Han S.T., Wu C.C., Chen K.F. (2020). Current Evidence and Limitation of Biomarkers for Detecting Sepsis and Systemic Infection. Biomedicines.

[83] Póvoa P., Coelho L., Dal-Pizzol F. (2023). How to use biomarkers of infection or sepsis at the bedside: Guide to clinicians. Intensive Care Med..

[84] Guarino M., Perna B., Cesaro A.E., Maritati M., Spampinato M.D., Contini C., De Giorgio R. (2023). Update on Sepsis and Septic Shock in Adult Patients: Management in the Emergency Department. J. Clin. Med..

[85] Barichello T., Generoso J.S., Singer M. (2022). Biomarkers for sepsis: More than just fever and leukocytosis—A narrative review. Crit. Care.

[86] Pierrakos C., Velissaris D., Bisdorff M. (2020). Biomarkers of sepsis: Time for a reappraisal. Crit. Care.

[87] Li Y., Wu Y., Gao Y. (2022). Machine-learning based prediction of prognostic risk factors in patients with invasive candidiasis Infection and bacterial bloodstream infection: A singled centered retrospective study. BMC Infect. Dis..

[88] Iwase S., Nakada T.A., Hattori N., Takahashi W., Takahashi N., Aizimu T., Yoshida M., Morizane T., Oda S. (2019). Interleukin-6 as a diagnostic marker for infection in critically ill patients: A systematic review and meta-analysis. Am. J. Emerg. Med..

[89] Shi J., Zhuo Y., Wang T.Q. (2024). Procalcitonin and C-reactive protein as diagnostic biomarkers in COVID-19 and Non-COVID-19 sepsis patients: A comparative study. BMC Infect. Dis..

[90] Velissaris D., Zareifopoulos N., Karamouzos V., Karanikolas E., Pierrakos C., Koniari I., Karanikolas M. (2021). Presepsin as a Diagnostic and Prognostic Biomarker in Sepsis. Cureus.

[91] Jolly L., Carrasco K., Salcedo-Magguilli M. (2021). sTREM-1 is a specific biomarker of TREM-1 pathway activation. Cell Mol. Immunol..

[92] Rashwan N.I., Hassan M.H., Mohey El-Deen Z.M., Ahmed A.E.A. (2019). Validity of biomarkers in screening for neonatal sepsis—A single center –hospital based study. Pediatr. Neonatol..

[93] Ruiz-Rodriguez J.C., Plata-Menchaca E.P., Chiscano-Camón L., Ruiz-Sanmartin A., Pérez-Carrasco M., Palmada C., Ribas V., Martínez-Gallo M., Hernández-González M., Gonzalez-Lopez J.J. (2022). Precision medicine in sepsis and septic shock: From omics to clinical tools. World J. Crit. Care Med..

[94] Sharma V., Grover R., Priyadarshi M., Chaurasia S., Bhat N.K., Basu S., Singh P. (2023). Point-of-Care Serum Amyloid A as a Diagnostic Marker for Neonatal Sepsis. Indian J. Pediatr..

[95] Ahuja N., Mishra A., Gupta R., Ray S. (2023). Biomarkers in sepsis-looking for the Holy Grail or chasing a mirage!. World J. Crit. Care Med..

[96] Buendgens L., Yagmur E., Ginsberg A., Weiskirchen R., Wirtz T., Jhaisha S.A., Eisert A., Luedde T., Trautwein C., Tacke F. (2020). Midregional Proadrenomedullin (MRproADM) Serum Levels in Critically Ill Patients Are Associated with Short-Term and Overall Mortality during a Two-Year Follow-Up. Mediat. Inflamm..

[97] Mickiewicz B., Thompson G.C., Blackwood J., Jenne C.N., Winston B.W., Vogel H.J., Joffe A.R. (2018). Biomarker Phenotype for Early Diagnosis and Triage of Sepsis to the Pediatric Intensive Care Unit. Sci. Rep..

[98] Li Q., Sun M., Zhou Q., Li Y., Xu J., Fan H. (2023). Integrated analysis of multi-omics data reveals T cell exhaustion in sepsis. Front. Immunol..

[99] Madhav A., Bousfield R., Pereira-Dias J., Cormie C., Forrest S., Keane J., Kermack L., Higginson E., Dougan G., Spiers H. (2024). A metagenomic prospective cohort study on gut microbiome composition and clinical infection in small bowel transplantation. Gut Microbes.

[100] Deng H.F., Sun M.W., Wang Y., Zeng J., Yuan T., Li T., Li D.H., Chen W., Zhou P., Wang Q. (2021). Evaluating machine learning models for sepsis prediction: A systematic review of methodologies. iScience.

[101] Shimabukuro D.W., Barton C.W., Feldman M.D., Mataraso S.J., Das R. (2017). Effect of a machine learning-based severe sepsis prediction algorithm on patient survival and hospital length of stay: A randomised clinical trial. BMJ Open Respir. Res..

[102] Alanazi A., Aldakhil L., Aldhoayan M., Aldosari B. (2023). Machine Learning for Early Prediction of Sepsis in Intensive Care Unit (ICU) Patients. Medicina.

[103] SepsisWatch. https://dihi.org/project/sepsiswatch/.

[104] Yang H.S. (2024). Machine Learning for Sepsis Prediction: Prospects and Challenges. Clin. Chem..

[105] Goto T., Kudo D., Uchimido R., Hayakawa M., Yamakawa K., Abe T. (2022). Web-based application for predicting the potential target phenotype for recombinant human thrombomodulin therapy in patients with sepsis: Analysis of three multicentre registries. Crit. Care.

[106] Adams R., Henry K.E., Sridharan A., Soleimani H., Zhan A., Rawat N. (2022). Prospective, multi-site study of patient outcomes after implementation of the TREWS machine learning-based early warning system for sepsis. Nat. Med..

[107] Liu D., Langston J.C., Prabhakarpandian B., Kiani M.F., Kilpatrick L.E. (2024). The critical role of neutrophil-endothelial cell interactions in sepsis: New synergistic approaches employing organ-on-chip, omics, immune cell phenotyping and in silico modeling to identify new therapeutics. Front. Cell. Infect. Microbiol..

[108] Yang Q., Wijerathne H., Langston J.C., Kiani M.F., Kilpatrick L.E. (2021). Emerging Approaches to Understanding Microvascular Endothelial Heterogeneity: A Roadmap for Developing Anti-Inflammatory Therapeutics. Int. J. Mol. Sci..

[109] Feaugas T., Sauvonnet N. (2021). Organ-on-chip to investigate host-pathogens interactions. Cell. Microbiol..

[110] https://cordis.europa.eu/project/id/258759/reporting/pl.

[111] https://www.fda.gov/medical-devices/in-vitro-diagnostics/nucleic-acid-based-tests.

[112] Gil Rosa B., Akingbade O.E., Guo X., Gonzalez-Macia L., Crone M.A., Cameron L.P., Freemont P., Choy K.-L., Güder F., Yeatman E. (2022). Multiplexed immunosensors for point-of-care diagnostic applications. Biosens. Bioelectron..

[113] Chen F., Hu Q., Li H., Xie Y., Xiu L., Zhang Y., Guo X., Yin K. (2023). Multiplex Detection of Infectious Diseases on Microfluidic Platforms. Biosensors.

[114] Garvey M. (2023). Antimicrobial Peptides Demonstrate Activity against Resistant Bacterial Pathogens. Infect. Dis. Rep..

[115] Gallardo-Becerra L., Cervantes-Echeverría M., Cornejo-Granados F. (2024). Perspectives in Searching Antimicrobial Peptides (AMPs) Produced by the Microbiota. Microb. Ecol..

[116] Schnell L., Felix I., Müller B., Sadi M., von Bank F., Papatheodorou P., Barth H. (2019). Revisiting an old antibiotic: Bacitracin neutralizes binary bacterial toxins and protects cells from intoxication. FASEB J..

[117] Castillo-Juárez I., Blancas-Luciano B.E., García-Contreras R., Fernández-Presas A.M. (2022). Antimicrobial peptides properties beyond growth inhibition and bacterial killing. PeerJ.

[118] Korbmacher M., Fischer S., Landenberger M., Papatheodorou P., Aktories K., Barth H. (2020). Human α-Defensin-5 Efficiently Neutralizes Clostridioides difficile Toxins TcdA, TcdB, and CDT. Front. Pharmacol..

[119] Schroom A.B., Paulowski L., Kaconis Y., Gutsmann T. (2021). Cathelicidin and PMB neutralize endotoxins by multifactorial mechanisms including LPS interaction and targeting of host cell membranes. Biophys. Comput. Biol..

[120] Leite M.L., Duque H.M., Rodrigues G.R., da Cunha N.B., Franco O.L. (2023). The LL-37 domain: A clue to cathelicidin immunomodulatory response?. Peptides.

[121] Nagaoka I., Tamura H., Reich J. (2020). Therapeutic Potential of Cathelicidin Peptide LL-37, an Antimicrobial Agent, in a Murine Sepsis Model. Int. J. Mol. Sci..

[122] Ciornei C., Egesten A., Bodelsson M. (2003). Effects of human cathelicidin antimicrobial peptide LL-37 on lipopolysaccharide-induced nitric oxide release from rat aorta in vitro. Acta Anaesthesiol. Scand..

[123] Nagaoka I., Hirota S., Niyonsaba F. (2002). Augmentation of the lipopolysaccharide-neutralizing activities of human cathelicidin CAP18/LL-37-derived antimicrobial peptides by replacement with hydrophobic and cationic amino acid residues. Clin. Diagn. Lab. Immunol..

[124] Inomata M., Horie T., Into T. (2020). Effect of the Antimicrobial Peptide LL-37 on Gene Expression of Chemokines and 29 Toll-like Receptor-Associated Proteins in Human Gingival Fibroblasts Under Stimulation with Porphyromonas gingivalis Lipopolysaccharide. Probiotics Antimicrob. Proteins.

[125] Pinilla G., Coronado Y.T., Chaves G., Muñoz L., Navarrete J., Salazar L.M., Taborda C.P., Muñoz J.E. (2022). In Vitro Antifungal Activity of LL-37 Analogue Peptides against *Candida* spp.. J. Fungi.

[126] Alshaya A.I., Bin Saleh K., Aldhaeefi M., Baderldin H.A., Alamody F.O., Alhamdan Q.A., Almusallam M.A., Alshaya O., Al Sulaiman K., Alshareef S. (2022). Colistin Loading Dose in Septic Patients with Gram Negative Infections. Infect. Drug Resist..

[127] Ambreen G., Salat M.S., Hussain K. (2020). Efficacy of colistin in multidrug-resistant neonatal sepsis: Experience from a tertiary care center in Karachi, Pakistan. Arch. Dis. Child..

[128] Berditsch M., Afonin S., Reuster J., Lux H., Schkolin K., Babii O., Radchenko D.S., Abdullah I., William N., Middel V. (2019). Supreme activity of gramicidin S against resistant, persistent and biofilm cells of staphylococci and enterococci. Sci. Rep..

[129] Swierstra J., Kapoerchan V., Knijnenburg A., van Belkum A., Overhand M. (2016). Structure, toxicity and antibiotic activity of gramicidin S and derivatives. Eur. J. Clin. Microbiol. Infect. Dis..

[130] Garvey M. (2022). Non-Mammalian Eukaryotic Expression Systems Yeast and Fungi in the Production of Biologics. J. Fungi.

[131] Garvey M. (2020). Bacteriophages and the One Health Approach to Combat Multidrug Resistance: Is This the Way?. Antibiotics.

[132] Gatea Kaabi S.A., Musafer H.K. (2020). New Phage cocktail against infantile Sepsis bacteria. Microb. Pathog..

[133] Singh A., Singh A.N., Rathor N., Chaudhry R., Singh S.K., Nath G. (2022). Evaluation of Bacteriophage Cocktail on Septicemia Caused by Colistin-Resistant Klebsiella pneumoniae in Mice Model. Front. Pharmacol..

[134] Li J., Shi H., Wang D., Lu Y., Zhang Z., Sun Y. (2014). Bacteriophage therapy for gut-derived sepsis caused by Acinetobacter baumannii in mice. Chin. J. Clin. Infect. Dis..

[135] Rahimi A., Soudi S., Vakilian S., Jamshidi-Adegani F., Sadeghizadeh M., Al-Hashmi S. (2023). Bacteriophage M13 Modulates The Sepsis-Related Inflammatory Responses And Organ Damage in A Clp Model. Shock Inj. Inflamm. Sepsis Lab. Clin. Approaches.

[136] Serwer P., Wright E.T., Lee J.C. (2019). High murine blood persistence of phage T3 and suggested strategy for phage therapy. BMC Res. Notes.

[137] Jennes S., Merabishvili M., Soentjens P., Win Pang K., Rose T., Keersebilck E., Soete O., Francois P.M., Teodorescu S., Verween G. (2017). Use of bacteriophages in the treatment of colistin-only-sensitive *Pseudomonas aeruginosa septicaemia* in a patient with acute kidney injury. Case Report. Crit. Care.

[138] Ujmajuridze A., Chanishvili N., Goderdzishvili M., Leitner L., Mehnert U., Chkhotua A., Kessler T.M., Sybesma W. (2018). Adapted bacteriophages for treating urinary tract infections. Front. Microbiol..

